# Defining the HIV Capsid Binding Site of Nucleoporin 153

**DOI:** 10.1128/msphere.00310-22

**Published:** 2022-08-30

**Authors:** Shunji Li, Jagdish Suresh Patel, Jordan Yang, Angela Marie Crabtree, Brenda Marilyn Rubenstein, Peik Karl Lund-Andersen, Frederick Marty Ytreberg, Paul Andrew Rowley

**Affiliations:** a Department of Biological Sciences, University of Idaho, Moscow, Idaho, USA; b Institute for Modeling Collaboration and Innovation, University of Idaho, Moscow, Idaho, USA; c Department of Chemistry, Brown University, Providence, Rhode Island, USA; d Department of Physics, University of Idaho, Moscow, Idaho, USA; e Center for Computational Molecular Biology, Brown University, Providence, Rhode Island, USA; University of Michigan Medical School

**Keywords:** HIV, NUP153, capsid, molecular modeling, FoldX, PyRosetta

## Abstract

The interaction between the HIV-1 capsid and human nucleoporin 153 (NUP153) is vital for delivering the HIV-1 preintegration complex into the nucleus via the nuclear pore complex. The interaction with the capsid requires a phenylalanine/glycine-containing motif in the C-terminus of NUP153 (NUP153C). This study used molecular modeling and biochemical assays to comprehensively determine the amino acids in NUP153 that are important for capsid interaction. Molecular dynamics, FoldX, and PyRosetta simulations delineated the minimal capsid binding motif of NUP153 based on the known structure of NUP153 bound to the HIV-1 capsid hexamer. Computational predictions were experimentally validated by testing the interaction of NUP153 with capsid using an *in vitro* binding assay and a cell-based TRIM-NUP153C restriction assay. This work identified eight amino acids from P1411 to G1418 that stably engage with capsid, with significant correlations between the interactions predicted by molecular models and empirical experiments. This validated the usefulness of this multidisciplinary approach to rapidly characterize the interaction between human proteins and the HIV-1 capsid.

**IMPORTANCE** The human immunodeficiency virus (HIV) can infect nondividing cells by interacting with the host nuclear pore complex. The host nuclear pore protein NUP153 directly interacts with the HIV capsid to promote viral nuclear entry. This study used a multidisciplinary approach combining computational and experimental techniques to comprehensively map the effect of mutating the amino acids of NUP153 on HIV capsid interaction. This work showed a significant correlation between computational and empirical data sets, revealing that the HIV capsid interacted specifically with only six amino acids of NUP153. The simplicity of the interaction motif suggested other FG-containing motifs could also interact with the HIV-1 capsid. Furthermore, it was predicted that naturally occurring polymorphisms in human and nonhuman primates would disrupt NUP153 interaction with capsid, potentially protecting certain populations from HIV-1 infection.

## INTRODUCTION

In eukaryotic cells, the nuclear envelope compartmentalizes the cytoplasm from the nucleoplasm. It is a physical barrier that must be traversed by viruses requiring access to the nucleus during their life cycle, particularly when infecting nondividing cells (1, 2). Access to the nucleus is through membranous pores in the nuclear envelope that are each stabilized by a large assemblage of ~30 different nucleoporin proteins called the nuclear pore complex (NPC) (3). The NPC regulates nucleocytoplasmic transport with a selectively permeable barrier of unstructured filamentous nucleoporins that fill the nuclear pore and project from its surface. These filamentous nucleoporins contain an abundance of phenylalanine/glycine (FG) repeats that create a hydrophobic barrier to prevent the free diffusion of large macromolecules. Cellular proteins interact directly with nucleoporins to enable the nuclear ingress and egress of specific cellular cargos. Interaction with FG nucleoporins is important for the efficient trafficking of macromolecules that are larger than ~40 kDa.

Lentiviruses require the NPC to transport viral proteins and nucleic acids during the infection of nondividing cells. Specifically, the HIV-1 genome is delivered to the NPC encapsidated in a fullerene cone constructed of monomeric capsid proteins (CA) assembled as hexamers and pentamers (4, 5). The assembled capsid has been observed docking with the surface of the NPC, but there is currently much debate on the exact mechanism of HIV-1 nuclear ingress (6). Many HIV-1 proteins have been shown to traffic to the nucleus, but the CA plays a dominant role in enabling the infection of nondividing cells (7, 8). Genome-wide RNA interference screens have identified several nucleoporins that are required for the completion of the HIV-1 life cycle (9–12). Of all the nucleoporins depleted from human cells in large-scale screens, NUP153 was consistently identified as being important for HIV-1 infection. NUP153 depletion results in up to a 100-fold drop in HIV-1 infectivity reducing nuclear import of cDNA and integration (13–17). The importance of NUP153 during viral nuclear ingress also extends to other primate lentiviruses (18). However, it is less critical for lentiviruses that infect other mammals, such as equine infectious anemia virus and feline immunodeficiency virus (16).

NUP153 has an overall disordered structure and is anchored by its N-terminal domain to the nuclear basket. The NUP153 C-terminal domain (NUP153C) is rich in FG motifs that can project into the cytoplasm and nucleoplasm (19). The FG-rich NUP153C is required for CA binding (20), with a motif at amino acid positions 1407 to 1423 (TNNSPSGVFTFGANSST) playing a dominant role in this interaction (16, 21). NUP153C interaction does not occur with CA monomers and is specific to a hydrophobic pocket at the interface between two adjacent monomers of CA in the assembled hexamer (21). Mutations within CA that disrupt this pocket can prevent HIV-1 infection of nondividing cells and nuclear ingress of the preintegration complex. The central FTFG sequence of the NUP153C motif (amino acids 1415 to 1418) is crucial for this interaction. Moreover, mutation of the amino acids F1415, T1416, and F1417 in NUP153 interferes with the CA hexamer interaction (16, 21). Similarly, other host proteins also interact with CA at the same hydrophobic interface as NUP153, including CPSF6 and SEC24C (22, 23). All these proteins insert phenylalanine sidechains into the same pocket but have differences in the surrounding amino acid sequence. CA-targeting small molecules PF74, BI-2, and Lenacapavir also insert phenyl groups into this pocket (24–26). Although phenylalanine residues are abundant in many nucleoporins, the sequence-specific determinants of the interaction between NUP153C and CA hexamers have not been rigorously determined.

This study aimed to use molecular modeling to comprehensively map the effect of all possible mutations at amino acid positions at the interface between NUP153 and the HIV-1 CA. Molecular modeling has been utilized in many biological systems to answer fundamental questions regarding protein folding and function (27–29) and provides detailed information about how protein residues interact with a binding partner at the atomic scale. There are 29 FG repeats within NUP153, and it is unclear what makes the C-terminal interaction motif unique in its specific interaction with CA. Molecular dynamics (MD) simulations and *in silico* mutagenesis were used to determine the residues required for CA interaction with NUP153. These modeling predictions were validated by assaying mutant NUP153 and its interaction with CA in cell-based and *in vitro* interaction assays. We found that modeling predictions correlated well with empirical studies. The stable interaction of specific residues of NUP153 with CA and their sensitivity to mutation enabled the determination of the specific sequence motif within NUP153 required for this important interaction that is required for HIV-1 replication.

## RESULTS

### Molecular modeling identified residues in NUP153 that are important for CA-binding.

**FIG 1:**
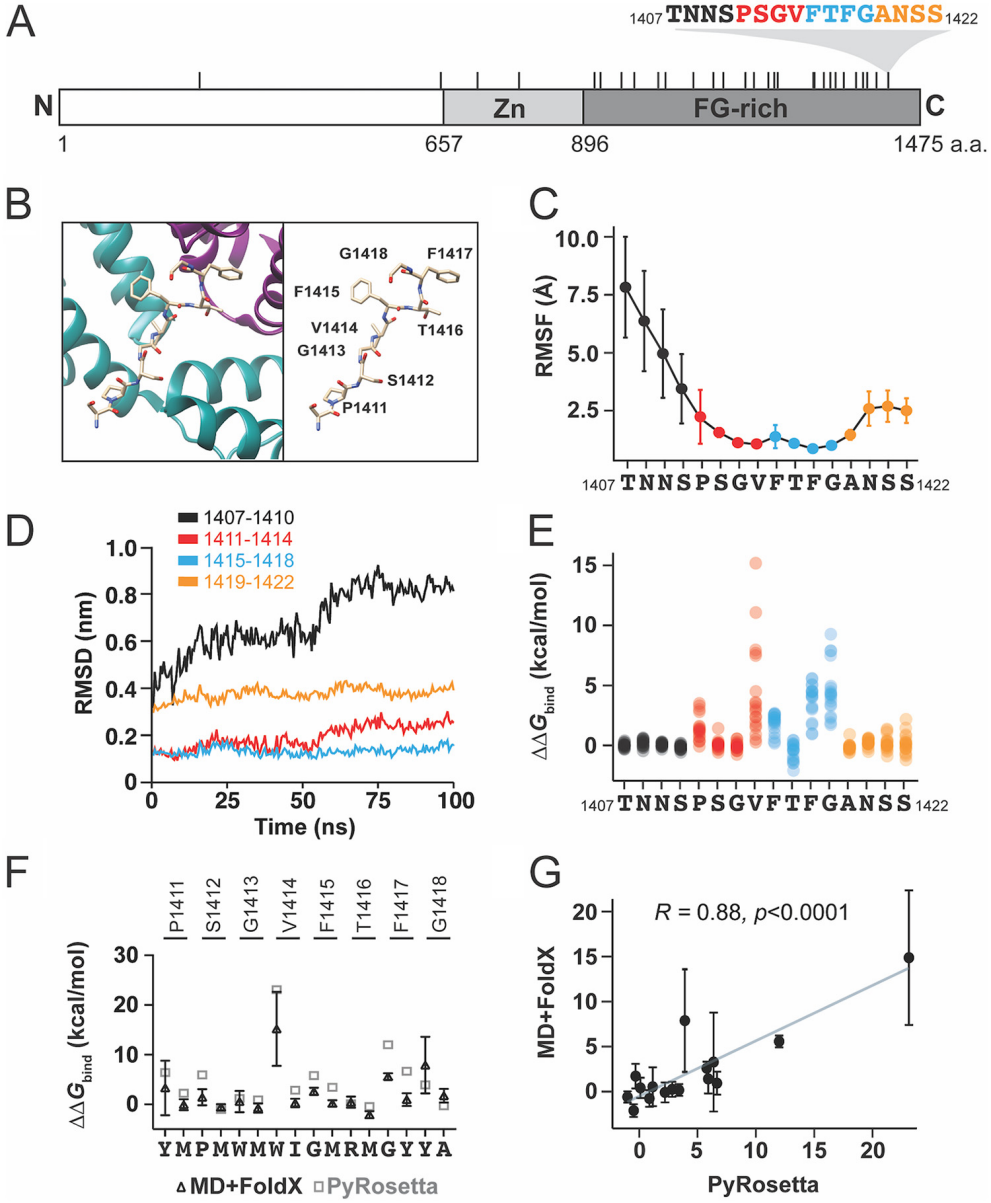
Molecular modeling of NUP153C-CA interaction defines a central eight amino acids that are stably associated with CA. (A) Domain diagram of the NUP153 protein. Tick marks represent FG repeats, with an expanded view of the FG motif that interacts directly with the HIV-1 CA (colored in relation to [C to E]). The numbering of NUP153 represents amino acid residues at the termini and domain boundaries. Zn, zinc finger domain. (B) A structural representation of the HIV-1 CA hexamer bound by the NUP153C peptide (PDB ID 4U0C) (left). Magenta, monomer A; green, monomer B. A labeled representation of NUP153C without the CA structure (right). (C) A plot of the average RMSF of each amino acid residue in six copies of the NUP153C residues 1407 to 1422 bound to the hexamer CA during a 100 ns MD simulation (*n* = 6, error bars are standard deviations). (D) RMSD of different regions of the NUP153C peptide during a 100 ns MD simulation. (E) ΔΔ*G*_bind_ was calculated by the MD+FoldX approach for all possible amino acid substitutions at each position in NUP153C (residues 1407 to 1422). (F) A comparison of the ΔΔ*G*_bind_ values calculated by MD+FoldX and PyRosetta for 16 NUP153C mutations. Error bars are standard deviations. (G) Correlation plot of ΔΔ*G*_bind_ estimated by MD+FoldX and PyRosetta, where the trendline shows the linear relationship between predicted ΔΔ*G*_bind_ values from the two different methods. Corresponding *R* and *P* values are displayed.

The FG-containing motif of NUP153 interacts with a hydrophobic pocket formed by two adjacent CA monomers (Fig. 1A and B) (21). The first molecular modeling approach involved conformational sampling of this CA-NUP153C complex via molecular dynamics (MD) simulations (30). Employing MD allowed the investigation of subtle conformational changes during the simulation and provided information on the stability of the interaction of NUP153C with CA. The X-ray crystal structure of the HIV-1 CA hexamer interacting with human NUP153C (PDB ID 4U0D) (21) was modified to better represent the wild-type CA hexamer bound to six chains of NUP153C (amino acids 1407 to 1422; Materials and Methods). The modified structure was subjected to MD simulation using the GROMACS software package to generate topology files and perform simulations (31, 32). The simulation was run for 100 ns, and snapshots were saved every 1 ns resulting in 100 snapshots for the protein complex (33). Root mean square fluctuations (RMSF) analysis of amino acids T1407-S1422 indicated that the region between P1411-G1418 fluctuated less while in the binding pocket of CA during MD simulation (RMSF < 2.5 Å) (Fig. 1C). Larger RMSF values of amino acids 1407 to 1410 and 1419 to 1422 indicated that they did not stably interact with CA during these simulations. The root mean square deviation (RMSD) calculated using the NUP153 peptide backbone atoms showed that amino acids P1411-A1419 were stably associated with CA hexamers compared to flanking sequences (T1407 to S1410 and N1420 to S1422) during 100 ns MD simulations (Fig. 1D). The protein-protein binding affinity prediction tools FoldX and PyRosetta were then employed to assess the effects of amino acid substitutions in NUP153C on the binding stability using the ΔΔ*G*_bind_ value, where ΔΔ*G*_bind_ = Δ*G*_bind_ (mutant) − Δ*G*_bind_ (wild-type). The FoldX analysis combined the FoldX software with MD simulations to compute 304 ΔΔ*G*_bind_ values for all possible 19 amino acid substitutions at each site in the NUP153C motif T1407-S1422 (Fig. 1E and Data Set S1) (27). Overall, a negative ΔΔ*G*_bind_ value suggested the binding is stabilized by an amino acid substitution, whereas a positive value indicates destabilization. Five amino acid residues that were stably associated with CA, P1411, V1414, F1415, F1417, and G1418, were considered critical binding sites for the CA interaction because substituting these residues resulted in more positive ΔΔ*G*_bind_ values (Fig. 1E). These residues were part of the region in NUP153C (P1411 to G1418) that was stably associated with CA during MD simulations (Fig. 1C and D). The ΔΔ*G*_bind_ values were compared to each substitution’s volume and hydrophobicity values (Table 1). Increased CA binding (decreasing ΔΔ*G*_bind_ values) was significantly correlated with the mutations that altered the volumes of the amino acid sidechains. For the residues F1415-F1417, increasing sidechain volume increased binding, whereas smaller sidechain volume at residues G1413, V1414, and G1418 increased binding (Table 1). CA binding at position T1416 was correlated with increased sidechain volume and hydrophobicity (Table 1).

**TABLE 1:** The volume of amino acid sidechains in NUP153C is more important for CA interaction than hydrophobicity^*a*^

Residue	Pearson correlation coefficient	
	Hydrophobicity	Vol
P1411	−0.368	0.4068
S1412	−0.4023	−0.4348
G1413	−0.4193	0.4738^*b*^
V1414	−0.2894	0.6345^*c*^
F1415	−0.3076	−0.7852^*c*^
T1416	−0.6186^*b*^	−0.4828^*b*^
F1417	−0.4132	−0.7489^*c*^
G1418	−0.2615	0.7582^*c*^

a Pearson correlation coefficient values comparing the ΔΔ*G*_bind_ MD+FoldX against the hydrophobicity and volume of amino acid sidechains.

b *P* < 0.05.

c *P* < 0.01.

10.1128/msphere.00310-22.3 Data Set S1 ΔΔ*G*_bind_ values calculated by MD+FoldX for all mutations at positions T1407 to S1422 in NUP153C. Download Data Set S1, PDF file, 0.05 MB. Copyright © 2022 Li et al. 2022 Li et al. https://creativecommons.org/licenses/by/4.0/ This content is distributed under the terms of the Creative Commons Attribution 4.0 International license.

To validate the predictions made by MD+FoldX, two substitutions were selected at each position from P1411-G1418 for analysis with PyRosetta (Data Set S2). These 16 mutations represented substitutions with either high or low ΔΔ*G*_bind_ values. Overall, PyRosetta agreed with the MD+FoldX predictions for mutations at positions G1413, T1416, and G1418, and predicted larger ΔΔ*G*_bind_ values for the disruptive mutants of P1411, S1412, V1414, F1415, and F1417 (Fig. 1F). Comparing the predictions of MD+FoldX and PyRosetta resulted in a strong positive correlation (Pearson's correlation coefficient *r* = 0.88, *P* < 0.0001) (Fig. 1G).

10.1128/msphere.00310-22.4 Data Set S2 ΔΔ*G*_bind_ values calculated by PyRosetta for selected mutations in NUP153C. Download Data Set S2, PDF file, 0.02 MB. Copyright © 2022 Li et al. 2022 Li et al. https://creativecommons.org/licenses/by/4.0/ This content is distributed under the terms of the Creative Commons Attribution 4.0 International license.

### Molecular modeling predicted the effects of mutations in NUP153C on the CA interaction as measured by cosedimentation.

**FIG 2:**
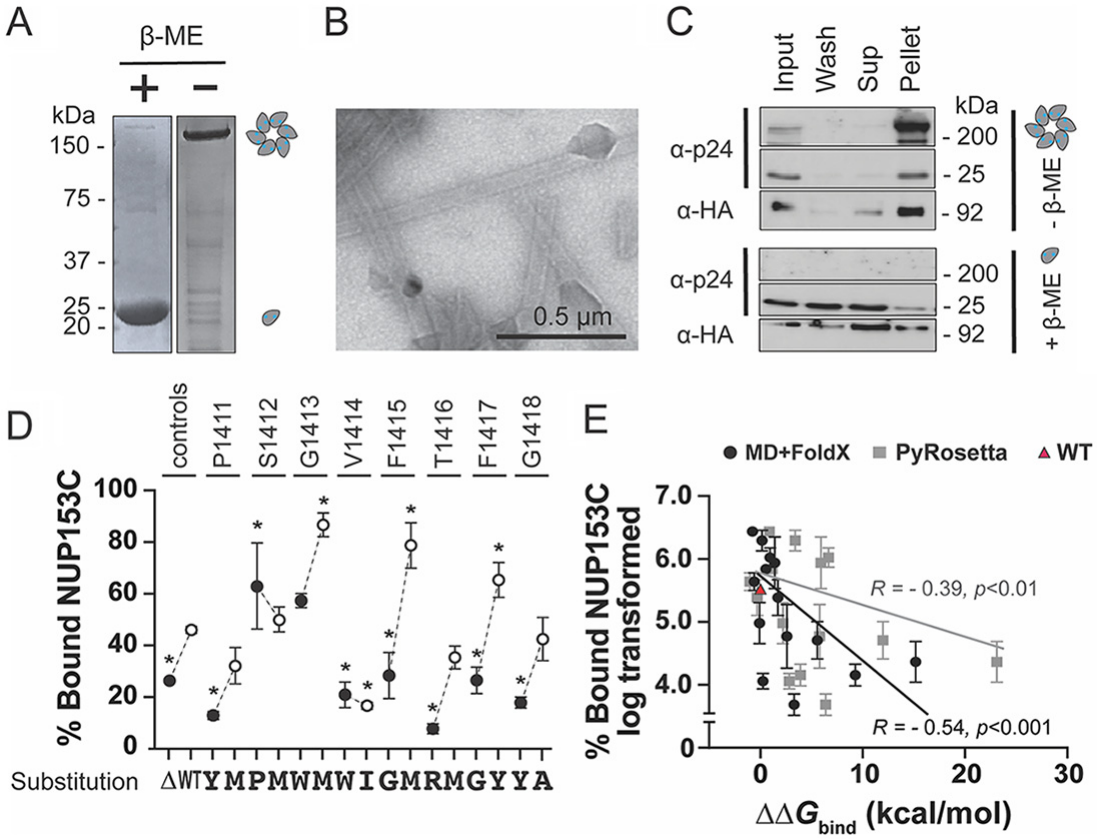
Molecular modeling predicts the effects of NUP153C mutations on CA interaction as measured by cosedimentation. (A) SDS-PAGE of the monomeric and the cross-linked hexameric CA. (B) Transmission electron micrograph of CA tubes assembled from cross-linked CA hexamers. (C) A Western blot of a cosedimentation assay using CA monomers (with the reducing agent β-mercaptoethanol [β-ME]) and CA tubes (without β-ME). Input, 10% of the total reaction volume. NUP153C was detected with an anti-HA antibody. (D) NUP153C mutants cosedimented with CA tubes. Western blot signals were normalized to input. Error bars are standard deviations. Control reactions included the wild-type NUP153C (WT) and NUP153C with a deletion of the CA interaction motif Δ1411-1418 (Δ). White data points represent mutations with low ΔΔ*G*_bind_ values, and black circles represent mutations with high ΔΔ*G*_bind_ values for each site. Statistical analyses were done using a one-way analysis of variance (ANOVA) followed by a Dunnett’s test. Asterisks indicate a significant difference from wild-type (*P* < 0.05). (E) Evaluation of the ΔΔ*G*_bind_ calculated by either MD+FoldX (black) or PyRosetta (gray) versus the experimental data set. The Pearson's correlation was calculated for each modeling method against three independent replicates of experimental data.

Molecular modeling predictions suggest that specific amino acids were more important for the NUP153C interaction with CA. To validate modeling predictions from both MD+FoldX and PyRosetta, 16 mutations in the central PSGVFTFG motif (residues 1411 to 1418) were created in NUP153C to represent eight substitutions with high ΔΔ*G*_bind_ and eight with low ΔΔ*G*_bind_. Each mutant NUP153C was expressed in a HEK293T human cell line as a TRIM domain fusion from the Rhesus Macaque TRIM5α restriction factor with a C-terminal HA tag. Cell lysates containing NUP153C were used to determine interaction with recombinant purified multimeric CA tubes (Fig. 2A and B). To assemble these tubes, CA monomers with engineered cysteine mutations were cross-linked to form hexamers (Fig. 2A) and assembled into higher-order structures (Fig. 2B). The NUP153C-CA interaction was determined based on the fraction of NUP153C that bound and cosedimented with multimeric CA tubes (Fig. 2C). Wild-type NUP153C efficiently bound to CA tubes, with 46% detected in the pellet fraction (Fig. 2C and D). The binding of NUP153C with CA depended on the assembly of the multimeric CA tubes and the formation of the NUP153 binding pocket because the reduction of the cystine bonds with β-mercaptoethanol disassembled the CA tubes and localized NUP153C to the supernatant fraction (Fig. 2C). A mutant NUP153C with a deletion of the entire interaction motif (ΔP1411-G1418) resulted in significantly less NUP153C binding to CA (Fig. 2D). As predicted by molecular modeling, the substitutions with low ΔΔ*G*_bind_ (P1411M, S1412M, T1416M, and G1418A) did not reduce the binding with CA compared to wild-type NUP153C (Fig. 2D). The mutations G1413M, F1415M, and F1417Y significantly increased the binding of NUP153C to CA tubes compared to the wild type. Disruptive mutations with high ΔΔ*G*_bind_ values generally reduced binding to CA tubes to the same extent as the NUP153 deletion mutant (ΔP1411-G1418) (Fig. 2D). S1412P and G1413W appeared to bind CA tubes *in vitro* similar to wild-type NUP153C (63% and 59%, respectively), reflecting their lower ΔΔ*G*_bind_ values compared to the other disruptive mutations (S1412P/G1413W: 1.413/0.544 kcal/mol MD+FoldX and 5.919/1.159 kcal/mol PyRosetta). The conservative mutation V1414I bound poorly to CA regardless of its lower ΔΔ*G*_bind_ values (0.26 kcal/mol MD+FoldX and 2.83 kcal/mol PyRosetta) (Fig. 2D). The results of these computational modeling approaches suggested that P1411, V1414, F1415, T1416, F1417, and G1418 are more important for CA interaction as they appeared most sensitive to mutation. S1412 or G1413 were less critical for CA interaction and could tolerate mutations as reflected in their overall lower ΔΔ*G*_bind_ values that were less than 2 kcal/mol (Fig. 1E). A comparison of all binding data with modeling predictions shows a significant negative correlation (Pearson's correlation coefficient −0.54 (*P* < 0.001) and −0.39 (*P* < 0.01), for MD+FoldX and PyRosetta, respectively) (Fig. 2E).

### Mutations predicted to disrupt NUP153C-CA interaction prevented HIV-1 restriction by TRIM-NUP153C.

**FIG 3:**
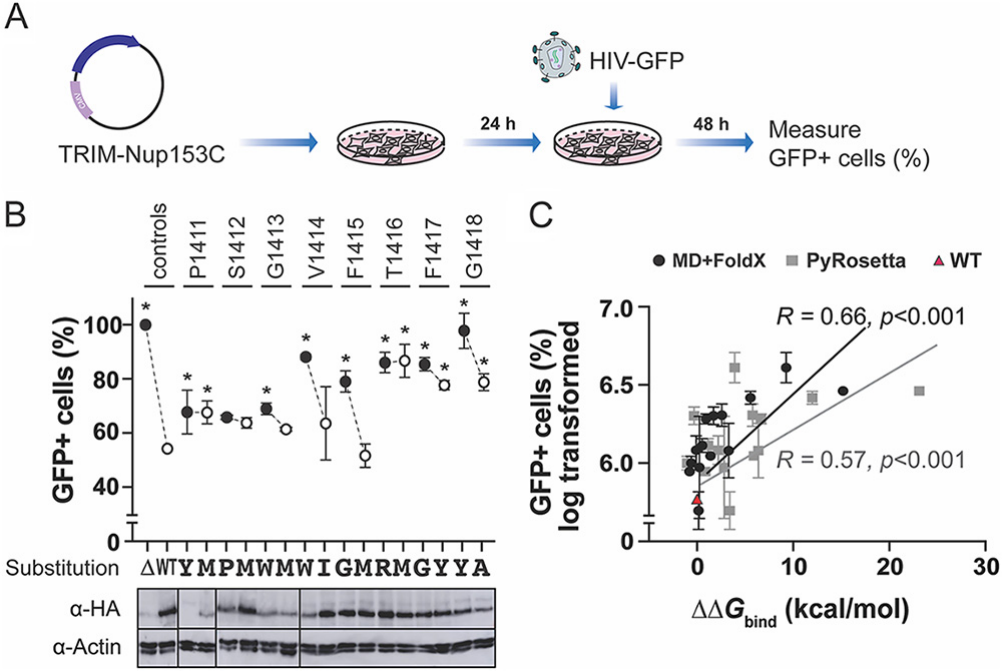
Molecular modeling predicts the effects of mutations in NUP153C on the CA interaction as measured by TRIM-NUP153C restriction. (A) A schematic representation of the workflow of a TRIM-NUP153C restriction assay. (B) NUP153C interaction with capsid was measured by the degree of HIV-1 restriction by transient expression of TRIM-NUP153C in HEK293T cells (top). The relative percentage of GFP-positive cells, indicating HIV-1 transduction, was measured by flow cytometry. White data points represent mutations that had low ΔΔ*G*_bind_ values, and black circles represent mutations that had high ΔΔ*G*_bind_ values for each site. Control reactions included the wild-type NUP153C (WT) and NUP153C with a deletion of the CA interaction motif Δ1411-1418 (Δ). Statistical analyses were done using a one-way ANOVA followed by a Dunnett’s test. Asterisks indicate significantly different from the interaction with wild-type NUP153 (WT) (*P* < 0.05). The expression of each mutant TRIM-NUP153C was examined by Western blotting (bottom). (C) The correlation of the empirical data presented in (B) with the ΔΔ*G*_bind_ calculated by either MD+FoldX or PyRosetta.

Modeling predictions made by MD+FoldX and PyRosetta were further scrutinized by testing the interaction between NUP153C and CA using a cell-based assay (Fig. 3A) (16). HEK293T cells were again transiently transfected with NUP153C with an N-terminal fusion to the TRIM domain. Cells expressing TRIM-NUP153C were challenged with HIV-GFP pseudotyped with VSV-G (Fig. 3A). Interaction between TRIM-NUP153C and the CA resulted in the restriction of viral replication and an ~2-fold drop in GFP-positive cells, to 53%, compared to the no TRIM control (Fig. 3B). Of the eight NUP153C mutants that were predicted not to affect CA interaction (low ΔΔ*G*_bind_ values), four displayed CA interaction that was not significantly different from wild-type, reducing HIV-1 transduction to an average of 60.43% (SD ± 7.98) (S1412M 64.06%, G1413M 61.71%, V1414I 63.87%, and F1415M 52.00%) (Fig. 3B). P1411M restriction was judged to be significantly different from wild-type but was still able to reduce transduction to 67.88% (SD ± 4.23). The remaining three mutations with low ΔΔ*G*_bind_ values were active in HIV-1 restriction but only reduced transduction to an average of 81.21% (SD ± 4.87) (T1416M 86.80%, F1417Y 77.86%, and G1418A 78.97%), indicating a loss of CA interaction. Importantly, seven of the eight disruptive mutations with high ΔΔ*G*_bind_ values significantly reduced transduction, with only S1412P judged to be similar to wild-type (Fig. 3B). Measuring the expression of the TRIM-NUP153C mutants indicated that the majority were expressed to a similar level within HEK293T cells (Fig. 3B, bottom). When there was reduced expression of NUP153C, there did not appear to be a reduction in HIV-1 restriction (P1411Y, P1411M, G1413W, G1413M). Of all the mutants tested, only V1414W showed decreased expression and a concomitant decrease in HIV-1 restriction. As demonstrated with the cosedimentation assay, we found a significant correlation between modeling predictions and TRIM-NUP153C restriction (Pearson's correlation coefficient 0.66 (*P* < 0.001) and 0.57 (*P* < 0.001) for MD+FoldX and PyRosetta, respectively) (Fig. 3C).

## DISCUSSION

Using molecular modeling we have identified the specific amino acid residues in the C-terminal domain of NUP153 that are important for CA interaction. We have determined that the minimal CA interaction motif is likely eight amino acids in length based on MD simulations and *in silico* mutagenesis using two different modeling software programs (MD+FoldX and PyRosetta). Specifically, we find that amino acids 1411 to 1418 (PSGVFTFG) remain stably associated with CA hexamers during MD simulations. Predictions made by both modeling approaches were in general agreement that the mutation of six of these eight residues would be more disruptive to CA hexamer interaction. An *in vitro* cosedimentation assay and a cell-based TRIM-NUP153C restriction assay were used to determine the biological relevance of the modeling predictions. Overall, MD+FoldX and PyRosetta predictions correlated significantly with empirical data, confirming the specific binding of CA hexamers to NUP153C. The validation of the molecular models enables the full use of all 304 mutational predictions to better understand the requirements for capsid interaction and to determine the effect of natural variation in NUP153, which is discussed below.

Validation of MD+FoldX ΔΔ*G*_bind_ predictions using PyRosetta highlights a strong agreement between two different tools using different energy functions. MD+FoldX seemed to discern CA-interacting from noninteracting mutants better than PyRosetta, as confirmed by experimental data (Fig. 2E and 3C). This difference between the models is likely because PyRosetta uses a single experimental structure of the NUP153-CA complex to predict ΔΔ*G*_bind_ values. In contrast, the MD+FoldX approach uses multiple snapshots extracted from MD simulations. Improved performance of MD+FoldX in predicting ΔΔ*G*_bind_ values also highlighted the importance of incorporating conformational sampling. Interestingly, both methods did poorly in predicting the effects of mutating T1416 in NUP153. Indeed, both methods suggested small ΔΔ*G*_bind_ values for T1416M and T1416R, indicating binding stabilization. Conversely, experimental data have shown that the mutations T1416M, T1416R (this study), and T1416A (21) disrupt CA interaction. This discrepancy between modeling and empirical data is likely due to the water-mediated interaction of T1416 with CA residues R173 and E63 (21). These modeling inaccuracies are expected because one of the significant limitations of fast protein-protein binding affinity prediction tools (FoldX and PyRosetta) is that they ignore the explicit presence of bridging water molecules (34).

The hydrophobic pocket in the CA hexamer has been reported to accommodate the host factors CPSF6 and SEC24C (21–23). The same pocket has also been targeted by small molecules such as PF74 and BI-2 (21, 24) and the antiviral drug Lenacapavir and its derivatives (25, 35). These different proteins and small molecules adopt slightly different conformations within the CA pocket, but with a common feature of a phenyl group interaction with CA. The phenyl group of F1417 is required for NUP153 interaction with CA, which overlays with CA-bound CPSF6 residue F321 and SEC24C residue F236. In addition, the backbone amides of F1417, F321, and F236 all form a hydrogen bond with the sidechain of CA N57. Mutations at position F1417 were overall predicted to be deleterious to CA interaction except for the conservative mutation F1417Y. The average ΔΔ*G*_bind_ value of all mutations at F1417 was 3.71 kcal/mol (SD ± 1.44), demonstrating that F1417 is crucial for CA interaction. Moreover, bulky sidechain substitutions were better tolerated at F1417, as demonstrated by a significant correlation between increasing sidechain size and CA interaction (Table 1). The importance of F1417 is confirmed by the empirical findings, except that the F1417Y substitution improved NUP153 *in vitro* binding to CA tubes. However, this result was not corroborated by the TRIM-NUP153 cell-based restriction assay. Similarly, modeling predicted that mutation of F1415 was also deleterious to CA interaction, but ΔΔ*G*_bind_ values were lower than that of F1417. Nevertheless, these data are consistent with the previous report that F1415A alone disrupts capsid interaction (16). The bulky F1415M mutation (0.17 kcal/mol MD+FoldX; 3.41 kcal/mol PyRosetta) was selected for empirical testing as it had the lowest ΔΔ*G*_bind_ value of any mutation at that position and was predicted to bind CA similar to wild-type NUP153. F1415M increased NUP153 binding to multimeric CA tubes but was similar to wild-type in the cell-based TRIM-NUP153 restriction assay. The positioning of the hydrophobic sulfur-containing sidechain of F1415M into the hydrophobic binding pocket between CA monomers is similar to the sulfonyl group of Lenacapavir (36). Both S1412 and G1413 were tightly anchored to CA during MD simulations but were insensitive to mutations based on modeling predictions, which was confirmed by empirical data. The ability of S1412 to tolerate mutation reflects the interaction with CA residue Q176 via the main chain. Similarly, G1413 can be substituted for tryptophan (G1413W) without altering CA interaction because the bulky sidechain would be exposed to the solvent. Even though these larger amino acids are accommodated at these positions, there is still a preference for small volume sidechains (Table 1). In several cases, it was observed that there are some differences in results between empirical assays, with NUP153 binding better to multimeric CA hexamer tubes. This could be due to NUP153 interaction with structurally uniform CA tubes *in vitro* as opposed to capsids derived from HIV-1 particles composed of pentamers and hexamers within human cells. Regardless, there is still a significant correlation between both empirical approaches and ΔΔ*G*_bind_ values derived from the two modeling methods.

Genetic variation in host factors hijacked by viruses can protect from viral infection and the *NUP153* gene is evolving rapidly in primates (37). However, there are no sites under positive selection in the CA interaction motif of NUP153 (37) and it is 100% identical across 35 primate species, except in gorilla (Gorilla gorilla) and drill (Mandrillus leucophaeus). The S1412P substitution in gorilla NUP153 would likely have a minor effect on CA interaction (ΔΔ*G*_bind_ 1.41 kcal/mol MD+FoldX). Drill NUP153 has a more deleterious G1418S substitution (ΔΔ*G*_bind_ 2.27 kcal/mol MD+FoldX). This substitution has a polar sidechain that is less likely to be accommodated within the hydrophobic CA pocket and limits the flexibility of the polypeptide backbone. Interestingly, a simian-human immunodeficiency virus (SHIV) chimera with the CA from SIV_mnd1_ (infecting Mandrillus sphinx) does not require NUP153 when infecting human cells (18). It is tempting to speculate that the incompatibility between SIV_mnd1_ and human NUP153 could be due to the adaptation of the SIV_mnd1_ CA to accommodate S1418 in the primate NUP153 or to circumnavigate NUP153 entirely. In humans, there were no high-frequency single nucleotide polymorphisms (SNPs) that would alter the amino acid sequence of the CA interaction motif in NUP153 (gnomAD database) (38). Two low-frequency nonsynonymous SNPs were found at the same positions as in gorilla and drill resulting in the mutations S1412A and G1418V. These mutations could potentially disrupt CA interaction and protect from HIV-1 infection (ΔΔ*G*_bind_ 0.09 and 3.29 kcal/mol MD+FoldX, respectively). Despite ongoing pressure from primate lentiviruses, there may have been selection against nonsynonymous mutations at the CA interacting FG-containing motif of NUP153.

The relatively small sequence required for CA interaction could mean that other FG repeats within NUP153 and other nucleoporins could bind to the HIV-1 capsid. This is supported by the fact that only a complete deletion of the FG-region of NUP153 will abolish CA binding (20) and nonsynonymous substitutions and small deletions do not completely perturb CA interaction (16). The relevance of other FG repeats in NUP153 for CA interaction remains to be thoroughly investigated.

## MATERIALS AND METHODS

### Molecular dynamics simulations.

The X-ray crystal structure of the HIV-1 CA hexamer interacting with a 17-mer peptide of human NUP153C was downloaded from Protein Data Bank (PDB ID 4U0D) (21) and used as a starting structure for MD simulations. To prepare the structure for molecular modeling, the 3D coordinates file was modified to remove all but six chains of CA monomer and six chains of NUP153C. MODELLER software altered engineered residues in CA protein to wild-type and built the missing residues in all the chains to complete the experimental structure (39). However, one of the missing residues, T1423, a terminal residue in NUP153C peptide could not be modeled correctly due to its random placement which led to clashes/overlap with the CA protein, hence it was not included. The complete structure of the HIV-1 CA hexamer bound to NUP153C (1407 to 1422, 16-mer) was subjected to MD simulation using the protocol reported in our previous study (33). Briefly, the AMBER99SB*-ILDNP force field and the GROMACS 5.1.2 software package were used for generating topology files and performing simulations (31, 32). The final production simulation was run for 100 ns, and snapshots were saved every 1 ns resulting in 100 snapshots for the protein complex. The MD trajectory was visualized using the VMD software package and analyzed using the *grmsf* module available in the GROMACS package to calculate the root mean square fluctuation (RMSF) of all the atoms in each residue in the NUP153 motif during the simulation (40).

### Mutagenesis of NUP153C by FoldX and PyRosetta.

MD snapshots of the HIV-1 hexamer CA − NUP153 complex were analyzed using the FoldX software to estimate the relative binding affinities (ΔΔ*G*_bind_) for all possible mutations at each site in the NUP153 motif. As with previous studies (27, 33), the FoldX analysis protocol involved processing each snapshot six times in succession using the RepairPDB command to energy minimize the snapshot and the BuildModel command to generate all possible 19 single mutations at each site in the NUP153C motif. The binding affinity (Δ*G*_bind_) was subsequently estimated using the AnalyseComplex command. ΔΔ*G*_bind_ for each mutation was calculated by taking the difference between mutated and wild-type Δ*G*_bind_ values. ΔΔ*G*_bind_ values were averaged across all individual snapshot estimates for each mutation. To estimate ΔΔ*G*_bind_ values for all possible 19 mutations at each amino acid site of NUP153C, 30,400 FoldX calculations were performed (16 NUP153C residues × 19 possible mutations at each site × 100 MD snapshots). Finally, 304 averaged ΔΔ*G*_bind_ values for all possible mutations of the NUP153 motif were calculated (Data Set S1). PyRosetta-4 was used to compute the difference in binding stability scores between 16 selected mutant and wild-type structures (PDB ID 4U0D) (41). The score is designed to capture the change in thermodynamic binding stability caused by the mutation (42). First, all sidechains sampled were repackaged from the 2010 Dunbrack rotamer library (43) and applied the quasi-Newton minimization method via the ‘dfpmin’ algorithm in PyRosetta (44) with a tolerance of 0.001 (45) and the REF2015 scoring function (46) and allowing both the backbone torsion and sidechain angles to move. This procedure was performed 10 times, and the lowest-scoring structure was selected for introducing mutations and subsequent binding stability calculations. Next, each missense mutation was introduced into the model of NUP153C. All residues within a 10 Å distance of the mutated residue’s center were repacked, followed by a Monte Carlo sampling coupled with a quasi-Newton minimization of the backbone and all sidechains. We performed 10 independent simulations of 5,000 Monte Carlo cycles each. To compute binding energy, the total energy of a bound state structure was scored, separated CA and NUP153C, and then scored the unbound state total energy. The binding energy (Δ*G*_bind_) was computed by subtracting the unbound state total energy from the bound state total energy. This procedure was performed 10 times, and the predicted ΔΔ*G*_bind_ was obtained by taking the average of the three lowest scoring structures. All molecular modeling data can be found in Data Set S1 and Data Set S2.

### Plasmids construction and mutagenesis.

The plasmid pLPCX-TRIM-NUP153C(human)-HA encoding the TRIM domain from TRIM5α of Rhesus macaque (residues 1 to 304) fused to the HA-tagged human NUP153 C-terminal domain (896 to 1475) was obtained from the Engelman laboratory. TRIM-NUP153C-HA was amplified by PCR and subcloned to the Gateway^TM^ entry vector pCR8 to create plasmid pUI034. Gateway^TM^ cloning introduced the gene into the custom destination vector pCDNA3-GW, following the manufacturer’s instructions (Thermo Fisher). Site-directed mutagenesis was performed using the Q5 site-directed mutagenesis kit, following the manufacturer’s instructions (New England Biolab). All primers used for cloning and site-directed mutagenesis can be found in Table S1, and a list of the plasmids used is in Table S2.

10.1128/msphere.00310-22.1 TABLE S1 Oligonucleotide primers used for cloning and site-directed mutagenesis. Download Table S1, PDF file, 0.1 MB. Copyright © 2022 Li et al. 2022 Li et al. https://creativecommons.org/licenses/by/4.0/ This content is distributed under the terms of the Creative Commons Attribution 4.0 International license.

10.1128/msphere.00310-22.2 TABLE S2 A description of the plasmids used in this study. Download Table S2, PDF file, 0.03 MB. Copyright © 2022 Li et al. 2022 Li et al. https://creativecommons.org/licenses/by/4.0/ This content is distributed under the terms of the Creative Commons Attribution 4.0 International license.

### Cells and viruses.

HEK293T cells (ACS-4500, ATCC) were maintained at 37°C with 5% CO_2_ in Dulbecco’s Modified Eagle Medium (Sigma-Aldrich number D6429) supplied with 10% Fetal Bovine Serum (Sigma-Aldrich), 2 mM l-glutamine (VWR number L0131-0100), and 1% Penicillin/Streptomycin solution (Corning, number 30–002). Single-cycle HIV-1 virus with a GFP reporter gene was generated using a 10 mm dish by cotransfecting HEK293T cells with 4 μg psPAX2 (Addgene number 12259), 4 μg pLJM1-EGFP (Addgene number 19319), and 4 μg pCMV-VSVG (Addgene number 8454) using Lipofectamine 3000 following the manufacturer’s instruction (Invitrogen). After 48 h, the supernatant was passed through a 0.45 μm filter and stored at −80°C.

### Purification and *in vitro* assembly of CA hexamers and tubes.

The expression of HIV-1 CA protein was adapted from Pornillos et al. (47). E. coli BL21(DE3)pLysS was transformed with pET11a-HIV-NL4-3 encoding CA with the mutations A14C and E45C. Transformed bacteria were cultured in LB media with ampicillin (15 μg/ml) and chloramphenicol (100 μg/mL) at 37°C until the optical density at 600 nm (OD_600_) was 0.8. The expression of CA was induced with a final concentration of 1 mM IPTG and incubated at 37°C for 4 h. Cells were centrifuged at 4,500 × *g* for 20 min at 4°C. Cells were suspended in lysis buffer (50 mL for 4 L; 50 mM Tris-Cl pH 8.0, 50 mM NaCl, 100 mM β-ME, protease inhibitor cocktail tablets [Sigma-Aldrich number 11836153001]). The cell suspension was incubated on ice for 20 min with the addition of 1 g of lysozyme and 50 U of Benzonase (EMD Millipore number 70746-3). The cell suspension was subjected to sonication (MICROSON XL Ultrasonic cell disruptor) for 10 s at 80% of maximum output power for a total processing time of 5 min. Between pulses, samples were allowed to cool for 30 s on ice. The cell lysate was clarified by centrifugation (27,000 × *g* for 1 h at 4°C) and incubated with supersaturated ammonium sulfate (final concentration 25% of the total volume) on ice for 20 min. Precipitated CA was collected by centrifugation at 9,000 × *g* for 20 min at 4°C. The pellet was suspended in dialysis buffer (20 mM MOPS pH 6.8, 20 mM β-ME), transferred to a 3.5K MWCO dialysis cassette (Thermo Fisher number PI66110), and dialyzed against 1 L dialysis buffer for 16 h.

CA was purified using ion-exchange chromatography (ÄKTA start protein purification system, Cytiva). Dialyzed lysate was centrifuged at 20,000 × *g* for 10 min at 4°C, passed through a 0.45 μm filter, and applied to a HiTrap SP FF (Cytiva number 17-5054-01) column connected to a HiTrap Q FF column (Cytiva number 17-5156-01). Fractions were collected at ~25% of the sodium chloride gradient (~0.25 M) and assayed for purity by SDS-PAGE. Eluted CA protein was dialyzed using a 10K MWCO cassette (Thermo Fisher number PI66130). To assemble CA oligomers, the cassette was sequentially incubated at 4°C in assembly buffer (25 mM Tris-Cl pH 8.0, 1 M NaCl) supplemented with 20 mM, 2 mM, and 0.2 mM β-ME for 8 h, 24 h, and 48 h, respectively. The efficiency of CA assembly into hexamers was assessed by SDS-PAGE, and assembly into multimeric tubes was confirmed by transmission electron microscopy (Franceschi Microscopy & Imaging Center, Washington State University).

### CA tube cosedimentation assay.

This assay was adapted from Selyutina et al. (48). Approximately 200,000 HEK293T cells were seeded into each well of a 12-well dish. After incubation for 24 h, cells were transfected with 500 ng of pCDNA3-TRIM-NUP153C using 1.5 μL of TransIT-293 transfection reagent (Mirus Bio). Cells were incubated for 24 h before harvesting by scraping into 100 μL of CA binding buffer (10 mM Tris, pH 7.4, 1.5 mM MgCl_2_, 10 mM KCl, 1× Halt protease and phosphatase inhibitor cocktail [Thermo Fisher number PI78440]). Cell lysates were mixed for 15 min at 4°C before centrifugation at 21,000 × *g* for 15 min at 4°C. Clarified cell lysates were collected, and the protein content was normalized to 1.5 mg/mL by Bradford assay. Next, 20 μL of CA tubes (~1.4 μM) and 80 μL of whole-cell lysate were mixed and incubated at room temperature for 1 h. The reaction was centrifuged for 8 min at 21,000 × *g* at 4°C. Next, 15 μL of the supernatant was collected and compared to samples that were not centrifuged using Western dot blotting.

### Western blotting.

Samples were separated by SDS-PAGE and transferred to nitrocellulose membranes by Trans-Blot Turbo Transfer System (1.0 A, 25V, 15 min). Alternatively, samples were loaded onto the nitrocellulose membrane using a Bio-Dot microfiltration apparatus (Bio-Rad). Membranes were blocked with 3% nonfat milk in TBS with 0.1% Tween 20 (TBST) for 1 h. To probe for the HA tag, the membrane was incubated with rat anti-HA-HRP (3F10, Sigma-Aldrich number 12013819001; 1 in 2,000 dilution) for 1 h. To probe for CA and actin, membranes were incubated with rat anti-p24 antibody (ARP-6457; NIH HIV Reagent Program; 1 in 5,000 dilution) or rat anti-actin antibody (clone C4, VWR number 10221-880; 1 in 500 dilution) for 1 h. Membranes were washed with 5 mL of TBST three times for 5 min each with gentle rocking. The anti-p24 blots were transferred to a new tray and probed against goat anti-rat antibody (Thermo Fisher number 62-652-0; 1 in 4,000 dilution) for 40 min. Blots were visualized, and signals were quantified using Amersham Imager 600. Exposure time was adjusted manually to 10 s for anti-HA blots, 4 s for anti-p24 blots, and 10 s for anti-actin blots.

### TRIM-NUP153C-mediated restriction and cell flow cytometry.

This assay was adapted from Matreyek et al. (16). 200,000 HEK293T cells were seeded and transfected with TRIM-NUP153C as described in the CA cosedimentation assay. Next, 24 h posttransfection, viral stocks with 8 μg/mL of Polybrene were titrated to achieve 30% GFP-positive cells for each lot of recombinant HIV-GFP. Media was discarded 24 h postransduction, and fresh medium was added to the wells. 48 h after transduction, cells were treated with 0.05% trypsin (VWR number 16777-202) and centrifuged at 2,000 × *g* for 3 min at room temperature. Cell pellets were suspended, fixed with 300 μL of Dulbecco's phosphate-buffered saline (DPBS; VWR number 45000-434) containing 1% paraformaldehyde (Electron Microscopy Sciences number 15710), and incubated at 4°C for 1 h. Cells were centrifuged at 2,000 × *g* for 3 min, and the cell pellet was suspended in 500 μL DPBS. This step was repeated twice, and the final pellet was suspended in 100 μL flow cytometry buffer (DPBS with 4% FBS) and transferred to a 96-well U bottom assay plate (Celltreat number 229590). Fluorescent cells were quantified using the CytoFLEX S instrument (Beckman Coulter).
