# Supplementary material for: Defining the HIV Capsid Binding Site of Nucleoporin 153
Source: mSphere. 2022 Aug 30;7(5):e00310-22. doi: 10.1128/msphere.00310-22 (PMC9599535; doi:10.1128/msphere.00310-22)
Supplement: Data Set S1 [file msphere.00310-22-s0003.pdf]

		MD+FoldX
**Mutation**	**ddg bind (kcal/mol)**	**standard deviation (kcal/mol)**
T1407I	-0.021239	0.482314
T1407L	-0.101076	0.50499
T1407K	-0.170447	0.484495
T1407M	-0.332049	0.549834
T1407F	-0.240156	0.587503
T1407P	0.259712	0.478745
T1407S	0.006824	0.314678
T1407W	-0.105979	0.645549
T1407Y	-0.179532	0.621325
T1407A	0.070942	0.442742
T1407V	0.174867	0.423387
T1407R	-0.286887	0.524093
T1407N	0.033744	0.465579
T1407D	0.376694	0.586458
T1407C	0.089222	0.400001
T1407Q	-0.004503	0.458229
T1407E	0.247433	0.554372
T1407G	-0.026637	0.509147
T1407H	0.014908	0.559925
N1408I	-0.189304	0.779964
N1408L	-0.17935	0.745146
N1408K	0.118161	0.697178
N1408M	-0.453569	0.786802
N1408F	-0.074693	1.038971
N1408P	0.231022	0.827432
N1408S	0.444545	0.745931
N1408T	0.377943	0.742958
N1408W	-0.026617	1.108523
N1408Y	-0.023889	0.998042
N1408A	0.521948	0.747556
N1408V	0.242687	0.71336
N1408R	0.007969	0.740745
N1408D	0.622452	0.960548
N1408C	0.27011	0.599819
N1408Q	0.235803	0.674811
N1408E	0.527225	0.865524
N1408G	0.558239	0.799609
N1408H	0.35633	0.92942
N1409I	-0.125551	0.562458
N1409L	-0.137531	0.54968
N1409K	-0.011172	0.476752
N1409M	-0.316545	0.661132
N1409F	-0.073192	0.625138
N1409P	-0.019544	0.573583
N1409S	0.222781	0.565143
N1409T	0.190299	0.51482
N1409W	-0.047317	0.669128
N1409Y	-0.027762	0.576039
N1409A	0.29697	0.511973
N1409V	0.074574	0.482974

N1409R	-0.07621	0.518342
N1409D	0.223562	0.564587
N1409C	0.122466	0.471685
N1409Q	0.056971	0.463512
N1409E	0.123943	0.574275
N1409G	0.254979	0.556735
N1409H	0.146036	0.528752
S1410I	-0.296406	0.48075
S1410L	-0.340196	0.533201
S1410K	-0.275881	0.457949
S1410M	-0.578754	0.608653
S1410F	-0.401384	0.629923
S1410P	-0.07848	0.523437
S1410T	-0.066351	0.297115
S1410W	-0.31653	0.693687
S1410Y	-0.357829	0.820866
S1410A	0.042901	0.230176
S1410V	-0.144493	0.362897
S1410R	-0.216877	0.546492
S1410N	-0.034852	0.30949
S1410D	0.124242	0.396473
S1410C	-0.074961	0.267646
S1410Q	-0.124516	0.376377
S1410E	-0.011732	0.381304
S1410G	0.181051	0.301374
S1410H	-0.093918	0.551201
P1411I	0.358241	1.20131
P1411L	0.403769	1.382153
P1411K	1.013065	1.310848
P1411M	-0.083443	1.100942
P1411F	2.804359	4.731006
P1411S	1.385641	0.882017
P1411T	1.26862	1.06241
P1411W	3.495069	6.037348
P1411Y	3.291333	5.493309
P1411A	0.747118	0.517836
P1411V	0.31477	0.924668
P1411R	1.286368	1.660193
P1411N	1.355975	1.100101
P1411D	1.599844	1.11993
P1411C	0.712906	0.677716
P1411Q	1.136602	1.195544
P1411E	1.164043	1.109344
P1411G	1.515196	0.788828
P1411H	2.996066	3.512577
S1412I	-0.303526	0.713189
S1412L	-0.386137	0.649113
S1412K	-0.070661	0.551532
S1412M	-0.587499	0.629049
S1412F	-0.255673	0.744054
S1412P	1.413089	1.614353
S1412T	0.224122	0.566866

S1412W	-0.083803	0.826381
S1412Y	-0.133335	0.787706
S1412A	0.090485	0.498513
S1412V	0.059172	0.664569
S1412R	0.066016	0.607532
S1412N	0.109421	0.520508
S1412D	0.202336	0.578354
S1412C	-0.003424	0.476787
S1412Q	0.088382	0.543565
S1412E	0.096794	0.595166
S1412G	0.160047	0.43843
S1412H	0.373525	0.707506
G1413I	-0.100411	1.009903
G1413L	-0.410799	1.025717
G1413K	0.07411	1.088049
G1413M	-0.757159	0.927442
G1413F	0.04378	1.913697
G1413P	-0.474342	0.674484
G1413S	-0.263695	0.780534
G1413T	-0.010391	0.925007
G1413W	0.5442	2.153326
G1413Y	0.207341	1.954601
G1413A	-0.138643	0.668513
G1413V	-0.167846	0.816132
G1413R	0.288455	1.09621
G1413N	0.121681	0.96554
G1413D	-0.212927	1.008236
G1413C	-0.428467	0.656201
G1413Q	-0.142827	0.994467
G1413E	-0.182759	0.906735
G1413H	0.535451	1.671978
V1414I	0.260271	0.840773
V1414L	0.504833	1.253585
V1414K	4.493915	2.259744
V1414M	0.896459	1.522981
V1414F	7.959788	4.458363
V1414P	1.500555	0.849567
V1414S	2.857822	0.619187
V1414T	1.938708	0.595926
V1414W	15.19362	7.467686
V1414Y	11.08039	5.930788
V1414A	2.375871	0.394748
V1414R	7.494056	3.398253
V1414N	2.363573	0.791575
V1414D	3.003999	0.922111
V1414C	1.648301	0.434266
V1414Q	3.229212	1.421932
V1414E	3.499378	1.517307
V1414G	3.600151	0.462141
V1414H	7.688817	2.943862
F1415I	1.446937	0.819066
F1415L	1.00078	0.717059

F1415K	2.085202	0.733506
F1415M	0.173853	0.649826
F1415P	1.884531	0.747129
F1415S	2.67967	0.795162
F1415T	2.331764	0.772998
F1415W	0.330959	1.061236
F1415Y	0.636109	0.805945
F1415A	2.582252	0.728083
F1415V	1.948761	0.768387
F1415R	2.307973	0.881468
F1415N	2.489324	0.750192
F1415D	2.561585	0.855171
F1415C	2.266629	0.721209
F1415Q	2.126483	0.748778
F1415E	2.035708	1.027118
F1415G	2.597332	0.729008
F1415H	1.75188	0.596069
T1416I	-1.479554	0.727849
T1416L	-1.484685	0.61436
T1416K	0.06441	0.938477
T1416M	-2.097057	0.713439
T1416F	-1.198737	1.392481
T1416P	-1.158168	0.996422
T1416S	0.107906	0.565064
T1416W	-1.317893	2.014378
T1416Y	-0.47566	1.925982
T1416A	-0.097789	0.671317
T1416V	-0.848545	0.518114
T1416R	0.420218	1.124746
T1416N	-0.021384	0.775098
T1416D	-0.31643	0.757834
T1416C	-0.378775	0.713324
T1416Q	-0.359392	0.774686
T1416E	-0.410943	0.825592
T1416G	0.411653	0.696707
T1416H	0.144621	1.100535
F1417I	3.1155	1.053408
F1417L	1.728962	0.569678
F1417K	3.987923	0.513785
F1417M	0.987848	0.390923
F1417P	4.43155	1.185157
F1417S	5.071283	0.718569
F1417T	4.487887	0.889119
F1417W	1.846979	1.869069
F1417Y	0.937911	1.274675
F1417A	4.51008	0.575244
F1417V	3.010654	0.925913
F1417R	4.544044	1.115185
F1417N	4.219349	0.639203
F1417D	5.551508	0.695604
F1417C	4.080873	0.554024
F1417Q	4.082893	0.654426

F1417E	5.023006	0.742018
F1417G	5.577509	0.658268
F1417H	3.221724	0.532769
G1418I	4.240927	3.467017
G1418L	3.692206	3.397895
G1418K	4.485421	3.520272
G1418M	2.40574	2.94653
G1418F	7.502875	5.640528
G1418P	4.767336	2.620114
G1418S	2.272417	1.715088
G1418T	3.39577	2.58801
G1418W	9.271188	7.267097
G1418Y	7.891621	5.698638
G1418A	1.725118	1.362657
G1418V	3.292556	2.792519
G1418R	4.937586	3.777391
G1418N	4.368394	2.937833
G1418D	3.960094	2.845769
G1418C	1.890332	1.807922
G1418Q	4.169312	3.34699
G1418E	4.276472	3.18227
G1418H	7.882029	5.033398
A1419I	-0.35606	0.478303
A1419L	-0.285393	0.443533
A1419K	-0.394681	0.511596
A1419M	-0.615647	0.459269
A1419F	-0.243274	0.501928
A1419P	0.903828	1.533863
A1419S	-0.119723	0.489395
A1419T	-0.034316	0.508772
A1419W	-0.280553	0.59533
A1419Y	-0.208477	0.527209
A1419V	-0.087772	0.441675
A1419R	-0.558447	0.616099
A1419N	-0.18928	0.493583
A1419D	0.012697	0.487941
A1419C	-0.170569	0.389982
A1419Q	-0.287692	0.500019
A1419E	-0.202503	0.49913
A1419G	-0.02189	0.375271
A1419H	-0.05014	0.483712
N1420I	-0.128212	0.655625
N1420L	-0.283892	0.611602
N1420K	0.134629	0.665515
N1420M	-0.519919	0.71098
N1420F	0.118043	1.062004
N1420P	0.110546	0.713267
N1420S	0.554747	0.567471
N1420T	0.444492	0.591197
N1420W	0.22025	1.423602
N1420Y	0.479721	1.529409
N1420A	0.547759	0.542809

N1420V	0.137049	0.53618
N1420R	0.100619	0.77224
N1420D	0.351194	0.558685
N1420C	0.261975	0.499469
N1420Q	0.199442	0.56884
N1420E	0.167338	0.60755
N1420G	0.630246	0.590138
N1420H	0.553973	0.97034
S1421I	-0.40445	0.944144
S1421L	-0.508733	1.045599
S1421K	0.024921	1.205723
S1421M	-0.892832	1.117214
S1421F	0.476418	2.542868
S1421P	-0.04882	0.980807
S1421T	-0.055979	0.590457
S1421W	1.256212	3.338984
S1421Y	1.352063	3.382986
S1421A	0.041738	0.475393
S1421V	-0.243517	0.736785
S1421R	0.313805	1.510144
S1421N	0.172656	0.727319
S1421D	0.332609	0.810371
S1421C	-0.169346	0.514282
S1421Q	0.02201	1.01885
S1421E	-0.002895	1.051484
S1421G	0.330401	0.578501
S1421H	1.076919	2.394046
S1422I	-0.626066	1.090804
S1422L	-0.872418	1.231647
S1422K	0.211085	1.219406
S1422M	-1.217141	1.112981
S1422F	0.111517	2.639925
S1422P	-0.275601	0.832809
S1422T	-0.132354	0.721518
S1422W	1.438676	3.574621
S1422Y	0.881034	3.304407
S1422A	-0.280131	0.518096
S1422V	-0.759115	0.891892
S1422R	0.768525	1.515023
S1422N	0.266044	0.867249
S1422D	0.418522	0.851424
S1422C	-0.238773	0.619572
S1422Q	0.085751	0.987009
S1422E	-0.014076	1.163705
S1422G	0.207887	0.566789
S1422H	2.166071	3.407942
