# Supplementary material for: Defining the HIV Capsid Binding Site of Nucleoporin 153
Source: mSphere. 2022 Aug 30;7(5):e00310-22. doi: 10.1128/msphere.00310-22 (PMC9599535; doi:10.1128/msphere.00310-22)
Supplement: Data Set S2 [file msphere.00310-22-s0004.pdf]

	ΔΔG (kcal/mol)	Standard deviation (kcal/mol)
P1411Y	6.379563183	0.13621128
S1412P	5.919420475	0.065546644
G1413W	1.158614946	0.225331457
V1414W	23.11274699	0.161622501
F1415G	5.788684214	0.014610211
T1416R	0.101729587	0.010269631
F1417G	11.98853215	0.000424209
G1418Y	3.913970731	0.144975616
P1411M	2.212056208	0.065278831
S1412M	-1.004421234	0.012639083
G1413M	0.875685671	0.284423366
V1414I	2.829267598	0.187657383
F1415M	3.411940856	0.01493812
T1416M	-0.472948322	0
F1417Y	6.664851972	0.051256815
G1418A	-0.307954697	0
