# Supplementary material for: Defining the HIV Capsid Binding Site of Nucleoporin 153
Source: mSphere. 2022 Aug 30;7(5):e00310-22. doi: 10.1128/msphere.00310-22 (PMC9599535; doi:10.1128/msphere.00310-22)
Supplement: TABLE S1 [file msphere.00310-22-s0001.pdf]

Primer ID	Sequence (5'- -3')	Note	Direction
SL027	AAACAACAGTtatTCAGGAGTGTTCAC	P1411Y	Forward
SL028	GTGAAGTTGAAATTTGTAGTG	P1411Y	Reverse
SL029	TCCATCAGGAtggTTCACATTTGG	V1414W	Forward
SL030	CTGTTGTTTGTGAAGTTG	V1414W	Reverse
SL031	TCCATCAGGAtacTTCACATTTGGTGCAAATTC	V1414Y	Forward
SL032	CTGTTGTTTGTGAAGTTG	V1414Y	Reverse
SL033	ATCAGGAGTGgccACATTTGGTG	F1415A	Forward
SL034	GGACTGTTGTTTGTGAAG	F1415A	Reverse
SL035	AGGAGTGTTCatgTTTGGTGCAAATTCTAG	T1416M	Forward
SL036	GATGGACTGTTGTTTGTG	T1416M	Reverse
SL037	AGTGTTCACAgctGGTGCAAATTCTAG	F1417A	Forward
SL038	CCTGATGGACTGTTGTTTG	F1417A	Reverse
SL039	GTTCACATTTtggGCAAATTCTAGC	G1418W	Forward
SL040	ACTCCTGATGGACTGTTG	G1418W	Reverse
SL041	GTTCACATTTtatGCAAATTCTAGCACAC	G1418Y	Forward
SL042	ACTCCTGATGGACTGTTG	G1418Y	Reverse
SL063	AGTGTTCACAgctGGTGCAAATTCTAG	F1417A	Forward
SL064	CCTGATGGACTGTTGTTTG	F1417A	Reverse
SL065	AAACAACAGTtggTCAGGAGTGTTCAC	P1411W	Forward
SL066	GTGAAGTTGAAATTTGTAGTG	P1411W	Reverse
SL067	AAACAACAGTatgTCAGGAGTGTTCAC	P1411M; reverse use SL066	Forward
SL068	TCCATCAGGAatcTTCACATTTG	V1414I	Forward
SL069	CTGTTGTTTGTGAAGTTG	V1414I	Reverse
SL070	ATCAGGAGTGggcACATTTGGTG	F1415G	Reverse
SL071	GGACTGTTGTTTGTGAAG	F1415G	Reverse
SL072	ATCAGGAGTGgacACATTTGGTG	F1415D; reverse use SL071	Forward
SL073	ATCAGGAGTGatgACATTTGGTG	F1415M; reverse use SL071	Forward
SL074	AGTGTTCACAggtGGTGCAAATTCTAG	F1417G	Forward
SL075	CCTGATGGACTGTTGTTTG	F1417G	Reverse
SL076	AGTGTTCACAgatGGTGCAAATTC	F1417D; reverse use SL075	Forward
SL077	AGTGTTCACAatgGGTGCAAATTC	F1417M; reverse use SL075	Forward
SL078	GTTCACATTTgctGCAAATTCTAGC	G1418A	Forward
SL079	ACTCCTGATGGACTGTTG	G1418A	Reverse
SL080	CAACAGTCCAccaGGAGTGTTCA	S1412P	Forward
SL081	TTTGTGAAGTTGAAATTTGTAGTGC	S1412P	Reverse
SL082	CAACAGTCCAatgGGAGTGTTCAC	S1412M	Forward
SL083	TTTGTGAAGTTGAAATTTGTAG	S1412M	Reverse
SL084	CAGTCCATCAtggGTGTTCACATTTG	G1413W	Forward
SL085	TTGTTTGTGAAGTTGAAATTTG	G1413W	Reverse
SL086	CAGTCCATCAatgGTGTTCACATTTGG	G1413M; reverse use SL085	Forward
SL087	AGGAGTGTTCggaTTTGGTGCAAATTC	T1416G	Forward
SL088	GATGGACTGTTGTTTGTG	T1416G	Reverse
SL094	ATCAGGAGTGtccACATTTGGTG	F1415S; F1415S (reverse use SL34)	Forward
SL095	AGGAGTGTTCagaTTTGGTGCAA	T1416R	Forward
SL096	GATGGACTGTTGTTTGTGAAG	T1416R	Reverse
SL097	AGTGTTCACAtatGGTGCAAATTC	F1417Y; reverse use SL38	Forward
SL139	GCAAATTCTAGCACACCTG	pUI034 motif-1 KO	Forward
SL140	ACTGTTGTTTGTGAAGTTG	pUI034 motif-1 KO	Reverse
