# Supplementary material for: Defining the HIV Capsid Binding Site of Nucleoporin 153
Source: mSphere. 2022 Aug 30;7(5):e00310-22. doi: 10.1128/msphere.00310-22 (PMC9599535; doi:10.1128/msphere.00310-22)
Supplement: TABLE S2 [file msphere.00310-22-s0002.pdf]

Plasmid ID	Specified	Origin	Backbone
pSL024	pLPCX-TRIM-NUP153C(human)-HA Amp	Dr. Alan Engelman	pLPCX
pUI034	pCR8-TRIM-Nup153C (Human)	This paper	pCR8
pUI020	pCDNA3-TRIM-Nup153C (Human)	This paper	pCDNA3
pSL123	pCDNA3-TRIMNUP153c Motif-1 KO	This paper	pCDNA3
pSL128	pET11A-CA-089	Dr. Owen Pornillos	pET
pSL131	pSPax2	Addgene	pSPax2
pSL132	pLJM1-eGFP	Addgene	pLJM1
pSL148	pCMV-VSV-G	Addgene	N/A
pSL064	pCDNA3-TRIMNup153c F1415S	This paper	pCDNA3
pSL065	pCDNA3-TRIMNup153c T1416R	This paper	pCDNA3
pSL066	pCDNA3-TRIMNup153c F1417Y	This paper	pCDNA3
pSLm1	pCDNA3-TRIMNup153c P1411Y	This paper	pCDNA3
pSLm2	pCDNA3-TRIMNup153c V1414W	This paper	pCDNA3
pSLm3	pCDNA3-TRIMNup153c V1414Y	This paper	pCDNA3
pSLm4	pCDNA3-TRIMNup153c F1415A	This paper	pCDNA3
pSLm5	pCDNA3-TRIMNup153c T1416M	This paper	pCDNA3
pSLm7	pCDNA3-TRIMNup153c G1418W	This paper	pCDNA3
pSLm8	pCDNA3-TRIMNup153c G1418Y	This paper	pCDNA3
pSLm18	pCDNA3-TRIMNup153c F1417A	This paper	pCDNA3
pSLm19	pCDNA3-TRIMNup153c P1411W	This paper	pCDNA3
pSLm20	pCDNA3-TRIMNup153c P1411M	This paper	pCDNA3
pSLm21	pCDNA3-TRIMNup153c V1414I	This paper	pCDNA3
pSLm22	pCDNA3-TRIMNup153c F1415G	This paper	pCDNA3
pSLm23	pCDNA3-TRIMNup153c F1415D	This paper	pCDNA3
pSLm24	pCDNA3-TRIMNup153c F1415M	This paper	pCDNA3
pSLm25	pCDNA3-TRIMNup153c F1417G	This paper	pCDNA3
pSLm26	pCDNA3-TRIMNup153c F1417D	This paper	pCDNA3
pSLm27	pCDNA3-TRIMNup153c F1417M	This paper	pCDNA3
pSLm28	pCDNA3-TRIMNup153c G1418A	This paper	pCDNA3
pSLm29	pCDNA3-TRIMNup153c S1412P	This paper	pCDNA3
pSLm30	pCDNA3-TRIMNup153c S1412M	This paper	pCDNA3
pSLm31	pCDNA3-TRIMNup153c G1413W	This paper	pCDNA3
pSLm32	pCDNA3-TRIMNup153c G1413M	This paper	pCDNA3
